# Optimizing the Management of the Main Acute Infections in Pediatric ORL: Tonsillitis, Sinusitis, Otitis media

**DOI:** 10.1016/S1808-8694(15)31387-2

**Published:** 2015-10-17

**Authors:** Tania Maria Sih, Lucia Ferro Bricks

**Affiliations:** 1 Adjunct professor, Laboratório de Investigações Médicas (LIM), number 40, Faculdade de Medicina da Universidade de São Paulo (FMUSP); 2 Adjunct professor, Departamento de Pediatria, Faculdade de Medicina da Universidade de São Paulo (FMUSP)

**Keywords:** otitis media, sinusitis, tonsillitis

## Abstract

Sinusitis, acute otitis media and tonsillitis are very frequent in children. Most of these infections are caused by viruses, but are generally treated with antibiotics. Inappropriate use of antibiotics favors the selection, growth and spread of resistant bacteria; these bacteria colonize the airways and affect the entire community. With the emergence of antibiotic-resistant bacteria, respiratory infections have become more difficult to treat. Effective strategies are needed to restrict the use of antibiotics without harming children that truly need these drugs.

**Aim:**

to present a critical analysis of the results of randomized and controlled studies on clinical and laboratory criteria used in diagnosing and treating tonsillitis, sinusitis and otitis.

**Methods:**

a review of randomized and controlled studies about these conditions published in MEDLINE and SCIELO from 2000 to 2006.

**Conclusions:**

Given that most of these infections progress favorably without antibiotics, the use of these drugs should be avoided unless the child belongs to a high risk group for complications, or symptoms persist or worsen with despite symptomatic treatment. Physicians and laypersons should have better knowledge about the natural evolution of acute respiratory infections.

## INTRODUCTION

The emergence of resistant microorganisms against antibiotics is directly related with the excessive use of these drugs.^1^, ^2^, ^3^ Upper airway infections are very common in children; nursing babies may present up to 10 or 12 upper airway infections each year.^4^, ^5^, ^6^

Most cases of sinusitis, acute otitis media and tonsillitis are caused by viruses and their course is self-limited; however, it is common to see antibiotics used for treating these infections, especially in three situations: patients complaining of throat pain, children with hyperemic tympanic membranes or coughing with exudate, and sinus radiographs showing opacity. Lack of knowledge about the natural progression of upper airway infections, poor validation of the clinical history, the physical examination and epidemiological data are associated with the excessive use of antibiotics. There are also other equally important factors that lead to the abusive use of medication, such as the low predictive value of clinical signs to differentiate viral from bacterial tonsillitis, the absence of easy and low cost laboratory tests for diagnosing upper airway infections etiologically, difficulty in accessing medical services (which generates insecurity about the risk of complications), false expectations that antibiotics used in viral upper airway infections will avoid future complications, and difficulties in interpreting radiological exams, which may not help differentiate viral sinusitis from bacterial or allergic sinusitis.^4^, ^5^

In this review, the authors critically analyze the results of randomized and controlled studies about clinical and laboratorial criteria that are used for the diagnosis and treatment of tonsillitis, sinusitis and acute otitis media, based on the PUBMED and SCIELO databases.

## TONSILLITIS

Tonsillitis may be caused by a variety of microorganisms (Table 1); Group A beta-hemolytic streptococcus (or Group A Streptococcus pyogenes - GAS) is, however, the most common etiological agent. It may lead to suppurative complications and non-suppurative conditions such as rheumatic fever and acute glomerulonephritis. It has been estimated that 75% of tonsillitis cases in children aged from 2 to 10 years are caused by viruses; however, most of these cases are treated with antibiotics.^4^, ^5^, ^6^ Excessive use of antibiotics in the treatment of pharyngotonsillitis maybe due to the difficulty in differentiating viral tonsillitis from those caused by Group A beta-hemolytic streptococcus (GABHS) and the expectation that antibiotics may avoid complications such as rheumatic fever.^4^, ^5^, ^6^, ^7^

**Table 1:** Main causal agents in acute tonsillitis.

Virus	Bacteria	Others
Rhinovirus	*Streptococcus pyogenes Group A beta hemolytic streptococcus)*	*Mycoplasma pneumoniae*
Coronavirus	Groups C and G streptococcus	Chlamydias
Adenovirus *	Anaerobes	
Herpes virus types 1 and 2	*Neisseria gonorrheae*	
Parainfluenza virus	*Corynebacterium diphtheriae*	
Coxackievirus A	*Arcanobacter hemolyticum*	
Epstein Bar Virus **	Yersinias	
Citomegalovirus		
HIV		
Infuenza A and B		

Note: * more common in children aged below 3 years

** more common after age 3 years

A meta-analysis comprising 27 studies totaling 2,835 patients with throat pain revealed that antibiotic treatment reduced the risk of rheumatic fever by nearly 80% (RR 0.22; 95% CI 0.02 to 2.08) and was associated with a lower risk of glomerulonephritis. There were also fewer suppurative complications: acute otitis media (RR 0.30; 95% CI 0.15 to 0.58); acute sinusitis (RR 0.48; 95% CI 0.08 to 2.76); peritonsillary abscess (RR 0.15; 95% CI 0.05 to 0.47). A decrease in symptoms (pain and fever) was seen only after the third day in the antibiotic-treated group, when about half of the placebo-treated patients were already asymptomatic. After one week, 90% of patients treated or not with antibiotics were symptom-free; for each six patients treated with antibiotics only one had a decrease in symptoms by the third day of treatment. The impact of antibiotics on symptoms was more significant when only patients with positive oropharyngeal swabs for streptococci were analyzed.^7^

The main purpose of treating acute tonsillitis cases with antibiotics is to reduce the possibility of suppurative and non-suppurative complications associated with GABHS, and to decrease the transmission of this bacterium to the community. Although there are many clinical criteria for differentiating bacterial and viral tonsillitis (Table 2), their sensitivity for identifying bacterial tonsillitis remains low.

**Table 2:** Clinical features of bacterial tonsillitis.

-Age between 5 and 15 yearss
- High fever
- Intense inflammation of the pharynx and tonsils: pain, edema, and exudate
- Presence of painful and enlarged anterior cervical lymph nodes (> 1.0 cm)
- Absence of signs and symptoms suggesting a viral cause: conjunctivitis, hoarseness, diarrhea and coryza
- History of exposure to a patient with streptococcal disease or onset of condition at the end of winter and beginning of spring

In the Netherlands 156 children (aged 4 to 15 years) with at least two criteria suggesting bacterial infection and a complaint of throat pain for at least seven days were evaluated, and the effectiveness of three or seven days of penicillin therapy was assessed. Use of the antibiotic did not decrease the symptoms or the complication rate, regardless of whether the children had confirmed or unconfirmed GABHS infection. However, in 96 children (60%) that had positive oropharyngeal cultures for GABHS and that were not treated with antibiotics, there was a higher recurrence rate of tonsillitis. It may be possible that the low complication detection rate was associated with the small sample size and a low prevalence in that country of nephrogenic strains or those associated with rheumatic fever. Thus, an inadequate diagnosis and treatment of streptococcal tonsillitis may increase the risk in regions where there is a high prevalence of rheumatic fever.^8^

A study comprising 1,810 children in three countries was undertaken to assess the World Health Organization criteria for identifying children with GABHS tonsillitis; this study revealed that the percentage of GABHS tonsillitis ranged from 25.6% in Brazil to 42% in Croatia. Although the World Health Organization criteria were highly specific for this bacterium (94 to 97%), the sensitivity was lower than 12%. These authors concluded that more sensitive criteria are needed for identifying GABHS, especially in places where the incidence of rheumatic fever is high.^9^

In Sweden, the GABHS was found in oropharyngeal cultures in 53 of 169 (31%) children aged over 4 years with acute tonsillitis. Among children with all of the highly suggestive criteria for bacterial infection (24%), the sensitivity was 82%, the specificity was 96%, the positive predictive value was 90% and the negative predictive value was 93% for the antigen-detecting rapid strep test; for the positive clinical assessment, the sensitivity was 36%, the specificity was 97%, the positive predictive value was 83% and the negative predictive value was 77%.^10^

The prevalence of GABHS was 34% in a study done in Sao Paulo, which aimed to compare cultures, molecular hybridization and the rapid strep test for detecting the GABHS-antigen in 50 children aged from 1 to 12 years with acute pharyngotonsillitis. GABHS was found in 30% of cultures, in 25% of the samples tested with molecular hybridization, and in 26% of the fast antigen detection test; there was no statistically significant difference among these three methods.^11^

Another study of 81 subjects aged over 12 years found that the rapid strep test sensitivity and specificity were 93.9% and 68.7%, and that the negative and positive predictive values were 94.2 and 67.4%.^12^

Although tonsillary cultures are considered the gold standard for the final etiological diagnosis of GABHS tonsillitis (about 95% accuracy), this test is not easily available and its results are slow. The American Academy of Pediatrics recommends using fast detection diagnostic tests for the specific group of streptococcal carbohydrates (enzyme immunoassay and latex agglutination) to differentiate viral from bacterial tonsillitis; these tests provide prompt results and are sensitive and specific for the diagnosis of streptococcal tonsillitis. It is important to note that neither oropharyngeal cultures nor rapid strep tests for detecting streptococci are able to differentiate patients with disease from GABHS carriers.^5^, ^13^

In developed countries, rapid strep tests for detecting oropharyngeal streptococcal antigens are being used more and more, given their high sensitivity. In Brazil, although these test have been used in some private clinics, they are not available in most public health units; the diagnosis is thus still based only on clinical criteria. Since these criteria are not sensitive for detecting GABHS tonsillitis, and given the risk of rheumatic fever in developing countries, the aforementioned tests should be made available so that clinicians working in public healthcare may avoid using antimicrobials excessively.^4^, ^5^, ^6^, ^7^

Prompt treatment of GABHS is not essential for preventing rheumatic fever, since the incidence of this complication is low when therapy is initiated up to nine days following the onset of symptoms. Although there are few benefits in avoiding the symptoms of tonsillitis, early therapy drastically reduces the transmission of strains causing rheumatic fever or acute glomerulonephritis. The risk of GABHS transmission in closed environments (households or schools) is high (35%); this risk fall markedly within 24 hours of penicillin therapy. On the other hand, early treatment of streptococcal infection is associated with a lower serological response and an increased recurrence rate of this infection.^13^

Treating GABHS tonsillitis adequately is just as important as diagnosing this condition correctly. Unfortunately, this is not what occurs in practice. Many children with throat pain are given inappropriate antibiotics for eradicating GABHS from the oropharynx, or are treated in shorter than recommended periods or incorrect doses or dose intervals.^5^, ^6^, ^13^

Various antibiotics are able to eradicate GABHS; some of them may be given in shorter regimens and in single daily doses, which may increase compliance. However, S. pyogenes remains highly sensitive to penicillin and cephalosporins.^13^, ^14^, ^15^, ^16^

Although macrolides may facilitate the treatment of streptococcal infections, low-dose azithromycin regimens appear to be inadequate for treating GABHS tonsillitis; furthermore, macrolide resistance is directly associated with its use. It is advisable to restrict the use of these drugs to patients that are allergic to penicillin or that present recurring tonsillitis.^13^

A metanalysis of 19 studies comprising 4,626 patients showed that treatment with 60 mg/kg azithromycin was superior to 10 days of treatment with penicillin or cephalosporins. Failures in eradicating GABHS were five times more frequent with other antibiotics compared to azithromycin; but when azithromycin was given at a dose of 30 mg/kg per treatment, its effectiveness was three times lower compared with a ten day treatment with other antibiotics. A treatment of tonsillitis with azithromycin, at a dose of 60 mg/kg in children or 500 mg/day during three days in adults is superior to therapy with other antibiotics, such as penicillin or the cephalosporins; it also has the advantage of reducing compliance problems.^14^

Studies done in the USA and Europe have shown that cephalosporins are three to four times more effective than oral penicillin for eradicating S. pyogenes as demonstrated in the laboratory.^15^ A metanalysis comprising 47 studies with 11,426 patients compared cephalosporins during 10 days against penicillin during 10 days and demonstrated that in Europe the bacteriological failure rate with cephalosporins was 4.3 times lower, and in the USA the same failure rate was 2.7 times lower. Studies that compared short cephalosporin treatments (4 to 5 days) with the classical oral penicillin treatment for 10 days also showed lower bacteriological failure rates with cephalosporins, although decreases in the failure rate were lower than that seen when cephalosporins were given for 10 days (1.3 to 2.4).^16^

A study done in teenagers and adults with GABHS-cause tonsillitis revealed that clindamycin at a dose of 300mg BID was equally effective compared to the association amoxicillin/clavulanate.^17^

Although these results have shown that some of the recent antibiotics are equally or more effective than penicillin in the treatment of GABHS tonsillitis, it should be borne in mind that these drugs are more costly, and that cephalosporins and macrolides have a higher impact on the selection of resistant strains compared to betalactamic drugs.^18^

Betalactamic antibiotics remain the first choice of treatment for bacterial tonsillitis, given their lower cost and a lower prevalence of resistant S. pyogenes against these drugs.^13^, ^19^, ^20^, ^21^

Finally, it is important to underline the need for caution when using non-steroidal anti-inflammatory drugs in the treatment of children with fever; if symptoms remain, other causes should be sought, such as viral infection or atypical bacteria.^22^, ^23^

## SINUSITIS

The common cold is the most frequent infectious disease in children and adults; it often causes persistent nasal exudate over more than seven to ten days. The nasal mucosa is continuous with the paranasal cavities; viral infections may thus also lead to sinus disease. Acute bacterial sinusitis, which is one of the most frequent complications of the common cold, should always be suspected in children with coughing and purulent nasal exudate lasting more than 10 to 14 days or when fever persists or recurs after the fourth day of an upper airway infection.

Plain radiographs of the paranasal cavities are not useful for the diagnosis of acute bacterial sinusitis, since both the common cold and allergic conditions may cause paranasal cavity opacity on radiographs, which cannot be distinguished from the radiological findings of acute bacterial sinusitis. When acute bacterial sinusitis is suspected or when symptoms recur, the best exam is computed tomography.^2^, ^3^, ^4^, ^5^, ^6^

Although many children with coughing and persistent nasal exudate are treated with antibiotics, these drugs are often little effective in solving the problem. Some studies have shown that symptoms resolve in only one of eight treated children (95% IC - 5 to 29).^24^, ^25^

A recent randomized controlled study comprising 82 children with nasal symptoms and image exams (ultrasound and paranasal radiographs) suggesting sinusitis revealed that the efficacy of therapy with cefuroxime axetil (125mg, BID, 10 days) was similar to the placebo; no significant difference was found in the proportion of completely cured children after two weeks. Cefuroxemine reduced the symptoms in only 6% of cases (95% IC -16 to 29).^26^

As with tonsillitis, the choice of the best therapy regimen for children with suspected bacterial sinusitis varies in different places; while some recommend amoxicillin in usual dosages, other advocate using wide spectrum antibiotics, such as high dose amoxicillin and clavulanate, cephalosporins and macrolides.

An analysis of 33 studies revealed that clarithromycin, compared to amoxicillin and amoxicillin/clavulanate, had little added benefit in reducing the symptoms of rhinosinusitis (OR = 1.12). Although clarithromycin is effective and safe for treating rhinosinusitis, its higher cost compared to betalactamic antibiotics has to be taken into account. The same applies to hew drugs such as telithromycin, which has been shown to be effective in adults at a dose of 800mg, once a day, for five days.^27^, ^28^, ^29^

Other recommended treatments for acute sinusitis include hypertonic saline solution and topical nasal corticosteroids. Most of the studies that compared these approaches with antibiotics or placebo have been done in selected adult groups^1^, ^2^, ^5^, ^30^, ^31^

## ACUTE OTITIS MEDIA

Acute otitis media is one of the most common infections in infancy, and the main reason given for using antibiotics in children; almost all children have had at least one episode of acute otitis media by age 3 years. Of these, 20% will present multiple episodes. It has been estimated that about 80% of acute otitis media episodes in nursing babies are cased by bacteria, in particular three organisms: S. pneumoniae, non-typifiable H. influenzae and M. catarrhalis^32^, ^33^.

Amoxicillin has been considered the drug of choice for treating acute otitis media, based on pharmacokinetic and pharmacodynamic principles; it has an adequate spectrum, excellent penetration into the middle ear, low toxicity and low cost. However, due to the many cases of acute otitis media that resolve spontaneously, as well as concerns about the excessive use of antibiotics, in many regions the recommendation is not to treat acute otitis media with antibiotics unless children present high fever or persistent signs and symptoms after being given analgesic for 48 to 72 hours.^32^, ^33^, ^34^, ^35^, ^36^

In the Netherlands, only 31% of children with acute otitis media are given antibiotics, while in the USA and Australia this rate is over 95%.^33^

A metanalysis of eight studies comprising 2,287 children with acute otitis media found that antibiotics were not superior to placebo in relieving symptoms within the first 24 hours of treatment, but reduced pain in 30% of cases after two to seven days.^33^ As the clinical condition resolved spontaneously in 80% of untreated children, the estimated benefit of antibiotic use was only 7%; estimates showed that symptoms resolved in only one child out of 15 that were treated with antibiotics.^33^

Symptoms of acute otitis media resolve in 61% of children after 24 hours, and in 80% after two to three days,^36^ regardless of antibiotic treatment.

One of the most significant issues when assessing children with otalgia is to differentiate acute otitis media from otitis media with effusion, due to a lower complication rate. The diagnosis of acute otitis media is based on an abrupt onset of otalgia and fever with fluid in the middle ear (tympanic membrane bulging, decreased or absent tympanic membrane mobility, fluid level behind the tympanic membrane or acute otorrhea) and an erythematous membrane. The differential diagnosis between acute otitis media and otitis media with effusion is not always easy, especially when the physical exam is done of a feverish, irritable and crying child with an upper airway infection, since crying is also associated with tympanic membrane erythema.^5^, ^6^

Added to these concerns about the immediate consequences of acute otitis media (complications and recurrence), doubts persist about whether antibiotics affect the resolution of the middle ear effusion or if lack of treatment affects hearing. Most of the children presenting otitis media with effusion after acute otitis media progress well.^37^, ^38^

It has been estimated that 59% of middle ear effusions following an episode of acute otitis media resolve within one month, and 74% of these cases resolve within three months. In cases of otitis media with effusion of unknown duration, resolution is 28% after 3 months and 42% after 6 months; in cases of chronic otitis media with effusion, resolution is somewhat slower (26% after 6 months and 33% after 12 months).^37^

A recently published randomized study about the progression of acute otitis media revealed that resolution of the middle ear effusion in children with acute otitis media occurs, on average, 7.5 days after treatment with amoxicillin or cefuroxime axetil is started, and that the conditions resolves in 69% of children after 14 days. The effusion in children with unilateral acute otitis media resolves more rapidly (5 days) than in children with bilateral effusion (10 days), and that bilateralism of acute otitis media was strongly associated with an increased risk of failure of therapy after two weeks, regardless of which antibiotic was used.^38^

The results of a Canadian study designed to compare the effectiveness of amoxicillin (60 mg/kg/day) with placebo in children with acute otitis media revealed that the response was 92.8% after 14 days of antibiotic treatment, and 84.2% in the placebo group. Amoxicillin therapy was superior to placebo, but the effect was small (8.6%). Most of these children progress well without antibiotics; the frequency of pain and fever during the first two days in the antibiotic-treated group was lower.

Recently published studies have recommended a more careful approach in the treatment of acute otitis media (WASP - wait and see prescription approach).^32^, ^33^, ^34^, ^35^, ^36^, ^37^, ^38^, ^39^, ^40^

In another study, children aged from 6 months to 12 years with mild or moderate acute otitis media were randomized for prompt antibiotic treatment (n=112) or antibiotics only if symptoms persisted (n = 111). Family members and caretakers accepted well this type of guidance, and on day 12, 69% of antibiotic-treated children had normal tympanic membranes compared to 51% in the control group. Tympanometry was within normal limits in 25% of children in the antibiotic-treated group and in 10% of children in the control group. There were 16% fewer failures in children treated promptly with antibiotics, but 66% of children in the control group did not require antibiotics; the cost in the antibiotic-treated group was US$47 on average, against US$11 on average in the control group. Visits to the emergency room, school or work absenteeism and recurrences were identical in both groups. The authors concluded that the instruction of using antibiotics only if symptoms persisted, compared to prompt antibiotic use, reduced antibiotic use by 73%. Furthermore, prompt antibiotic use increased oropharyngeal colonization by multiresistant S. pneumoniae strains soon after cessation of treatment.^34^

Still another study comprised 283 children with acute otitis media (6 months to 12 years) randomized either to be given antibiotics only if symptoms persisted or worsened not relieved by ibuprofen (WASP approach) or to the classical approach of prompt antibiotic and analgesic therapy. The results revealed that 63% of children did not require antibiotics and had no complications in the WASP approach group. Medication use was associated with persistent fever or otalgia.^39^

Little et al. (2006)^40^ monitored 315 children aged from 6 months to 10 years during one year, which had been randomized into a prompt antibiotic therapy group and a conservative approach group (WASP, or giving antibiotics only if symptoms persisted or worsened regardless of analgesics) for the treatment of acute otitis media. The short-term, 3-month and 12-month progression was similar in both groups, except for children with two or more previous episodes of acute otitis media. Among these children there were more recurrences of otalgia in the first three months (39% vs. 10%), but the number of sequelae one year later was not different from the groups of children treated with antibiotics. These data suggest that prompt antibiotic therapy does not alter the progression of acute otitis media.^40^

The American Academy of Pediatrics (Table 3) recommends prompt antibiotic treatment in the following conditions:

1) children aged below six months;
2) children aged from 6 to 24 months with evident signs of acute otitis media, high fever and/or intense otalgia, or if the child cannot be reassessed if the condition worsens;
3) children with an underlying disease (immuno-compromised, with cleft palate, malformed);
4) children aged over 2 years whose symptoms do not regress after 48 to 72 hours of symptomatic therapy.

**Table 3:** Recommendations for using antibiotics in acute otitis media.

1.	Children under age 6 months, even if symptoms are not severe and fever is not high, due to the high complication risk;
2.	Children aged from 6 to 24 months with high fever (T≥ 390C within the last 24 hours) or severe otalgia or if a reassessment is not possible if the condition worsens;
3.	Children over age 24 months with fever and/or intense otalgia or if the condition persists or worsens after 48 to 72 hours of symptomatic therapy;
4.	Children with predisposing diseases for acute otitis media (cleft palate, genetic syndromes, immune deficiency).

Although many studies have shown that most children with otitis, not treated with antibiotics, improve rapidly and have no complications, criticism has been leveled at the more conservative approach:

1) inclusion and exclusion criteria in these studies are not always clear;
2) many studies have included few children aged under 2 years, exactly those more prone to complications;
3) not all children seek medical help within the first 24 hours of the onset of symptoms; it is not advisable to wait another 48 to 72 hours before starting antibiotics in symptomatic children;
4) it is not always possible to assure that children will return for a reevaluation, especially in emergency care units;
5) some studies have indicated that suppurative complications, such as mastoiditis, are more frequent in countries with more restrictions against using antibiotics for the treatment of acute otitis media; since this complication is rare, the sample size needs to be larger to assure that there are no differences in the progression of children with acute otitis media treated or not with antibiotics.^40^, ^41^, ^42^, ^43^

Antibiotic selection for treating acute otitis media is also controversial. While some authors have recommended therapy with high doses of amoxicillin (80 to 90 mg/kg/day), other authors have suggested that the usual doses (40 to 45 mg/kg/day) are enough for treating children living in communities in which the prevalence of penicillin-resistant S. pneumonia is low as well as in children over 6 months that have been given at least three doses of the heptavalent pneumococcal conjugated vaccine. In areas where the prevalence of antibiotic-resistant strains is high, use of wide-spectrum antibiotics, such as cephalosporins and macrolides, is becoming more and more common. Dosages and duration of treatment also vary; some studies have indicated that cephalosporins or azithromycin during 3 to 5 days may be as effective as 10 days of amoxicillin, with a higher probability of adhesion to the treatment.^13^, ^44^, ^45^, ^46^, ^47^ Many of these recommendations have been based on studies that used double tympanocentesis to assess whether the bacteria had been eradicated from the middle ear; furthermore, these studies have taken place in countries where the prevalence of bacterial resistance is high.

Unfortunately, the local patterns of antibiotic resistance of strains that colonize the airways of children remain unknown in most areas, making it difficult to select the best antibiotic regimen for treating acute otitis media. It is essential to bear in mind that all antibiotics may increase resistance when deciding upon their use; furthermore, wide-spectrum antibiotics, such as the macrolides and new cephalosporins, have a higher ecological impact and are more costly than amoxicillin.^18^

For these reasons, we believe that the best approach in the treatment of acute otitis media is to restrict antibiotic use in mild acute otitis media cases (low fever and little pain) as long as the child may be reassessed in the short-term if symptoms worsen or persist, and if necessary to initiate therapy with amoxicillin at usual doses. Exceptions would be children at risk for resistant infections (recent antibiotic treatment, recurring otitis, immune deficiency).

The use of other drugs to treat acute otitis media, such as decongestants, non-steroidal anti-inflammatory drugs, and topical nasal or oral corticosteroids, is also controversial. Most of these drugs appear to provide no benefit to children with acute otitis media, and may cause adverse events.^48^, ^49^ Although topical corticosteroids singly or with antibiotics may have some effect in reducing middle era effusions in the short term, there is not enough information to assess their long-term efficacy; they should thus be recommended only for children with signs and symptoms of atopic disease.^50^

## CONCLUSION

The clinical history, the physical exam, and epidemiological data are essential for the diagnosis of tonsillitis, sinusitis and otitis.

Rapid strep tests for detecting streptococcal antigens in the oropharynx should be made available in public and private healthcare units, given the poor specificity of clinical criteria for differentiating between viral and bacterial tonsillitis.

Most cases of acute bacterial sinusitis and acute otitis media progress favorably without antibiotics; the guideline of prescribing analgesics/antithermic medication and nasal hygiene should be the first measure in the treatment of these infections. This approach is only applicable to children aged over 6 months, with no co-morbidities or signs and symptoms of severe disease.

Physicians and lay persons need to be informed about the natural progression of upper airway infections and the risks of excess antibiotic use in children; at the same time, these children need to have prompt access to a reassessment if symptoms persist or worsen. In these situations, antibiotic treatment should be started immediately.

A surveillance system providing information about resistant patterns of S. pneumoniae and H. influenzae strains that colonize the airways and that cause acute otitis media in children is needed, since there are wide local and seasonal variations in the resistance to antibiotics.
